# A Practical Chemo-enzymatic Approach to Highly Enantio-Enriched 10-Ethyl-7,8-dihydro-*γ*-ionone Isomers: A Method for the Synthesis of 4,5-Didehydro-*α*-Ionone

**DOI:** 10.3390/ijms13055542

**Published:** 2012-05-09

**Authors:** Assem Barakat, Abdullah M. Al-Majid, Yahia Nasser Mabkhot, Zeid Abdullah Al-Othman

**Affiliations:** Department of Chemistry, Faculty of Science, King Saud University, P.O. Box 2455, Riyadh 11451, Saudi Arabia; E-Mails: amajid@ksu.edu.sa (A.M.A.-M.); yahia@ksu.edu.sa (Y.N.M.); zaothman@ksu.edu.sa (Z.A.A.-O.)

**Keywords:** timberol, ionone, bio-catalysis, lipase, asymmetric catalysis

## Abstract

An efficient and convenient strategy for the enantioselective synthesis of enantiomerically enriched 10-ethyl-7,8-dihydro-*γ*-ionone isomers (*R*)-(+)-**7**, and (*S*)-(−)-**7** are described utilizing a lipase mediated resolution protocol, and reductive elimination of the secondary allylic alcohol as the key step. The enantioselective and diastereoselective lipase kinetic acetylation of 4-hydroxy-*γ*-ionone derivatives **6a** afforded the 4-acetyl-*γ*-ionone derivatives (−)-**8**, and the 4-hydrox-*γ*-ionone derivatives (+)-**6a**, which are suitable precursors of the desired products. Stereospecific palladium-mediated elimination of allylic acetate provides the target compounds with an excellent enantiomeric excess and yield. Additionally, the novel 4,5-didehydro-*α*-ionone **13** is obtained from readily prepared (2,6,6-trimethylcyclohexa-2,4-dien-1-yl) methanol **9**. The structures of all newly synthesized compounds have been elucidated by ^1^H, ^13^C NMR, GC-MS, and IR spectrometry. These compounds represent a new class of odorants that may be of pivotal relevance in industrial perfumery.

## 1. Introduction

Timberol^®^ and Ionones are among the most important fragrance constituents due to their distinctive fine woody, amber, violet and rose scents [1,2]. The industrial creation of new perfumes [3,4] needs two essential lines of research: the discovery of new odorous molecules and the reinvestigation or chemical modification of older commercial products. Due to the unpredictable relationship between chemical structure and odor [5], the latter approach is particularly interesting from a chemical point of view. Indeed, many fragrances are sold as a mixture of isomers whose specific contribution to the perceived odor may be very different. Moreover, olfactory evaluation shows that the regioisomeric purity and the absolute stereochemistry of these compounds dramatically determines the fragrance properties, sometimes with amazingly pronounced differences between the notes and the odor thresholds of the isomers. Taking advantage of processes based on the enzyme-mediated resolution, Fuganti *et al*. [6] have reported a synthetic approach to the olfactory active components of the woody odorant timberol^®^ whereas Serra *et al*. [7,8] described the enantioselective synthesis of a number of natural odorants with the ionone skeleton. Furthermore, the exocyclic double bond confers particularly characteristic nuances to the fragrance, which can favorably complement other widely used compound from the same family [9–15].

**Figure 1:**
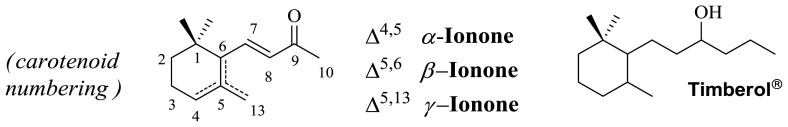
Timberol and Ionone framework.

In this context, we have focused our attention on the odorants in a combination of timberol and ionone framework (Figure 1) that are of pivotal relevance in industrial perfumery.

## 2. Results and Discussion

### 2.1. Preparation of 10-Ethyl-7,8-dihydro-γ-ionone

**Scheme 1:**
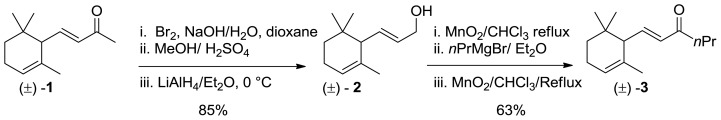
Synthesis of *α*-ionone isomer **3**.

We prepared compound **7** in racemic and enantiomer-enriched form starting from commercially available racemic *α*-ionone. It was introduced by Dragoco [16] as a synthetic fragrance with fixative properties with the brand name timberol^®^. Funganti *et al*. have previously developed a stereoselective procedure that allows the conversion of *α*-ionone derivatives into *γ*-ionone derivatives [17,18]. The regioselective base-mediated isomerization of 4,5-epoxy-4,5-dihydro-*α*-ionone followed by reductive elimination of the obtained allylic alcohols were the key steps of our syntheses. Therefore, we decided to apply the latter synthetic pathway for the conversion of compounds **1** into **7**. The synthesis began with the reaction of *α*-ionone **1** with Br_2_ and NaOH as base in a mixture of H_2_O and dioxane, the resulting intermediate acid was treated with MeOH in the presence of a catalytic amount of H_2_SO_4_ to afford the corresponding methyl ester. The later compound was reduced with LiAlH_4_ to produce the allylic alcohol **2**. The latter allylic alcohol was oxidized and the obtained aldehyde was treated with propyl magnesium bromide. The resulting ionol was then converted into pure **3** by an oxidation reaction using MnO_2_ (Scheme 1).

**Scheme 2:**
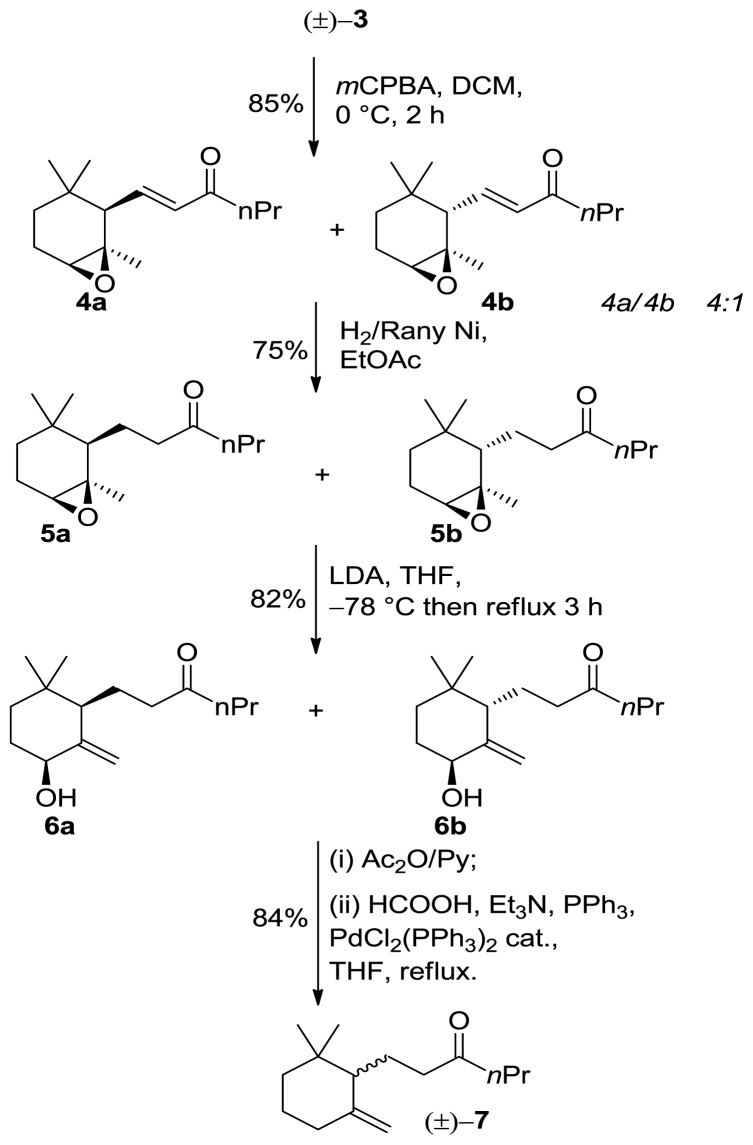
Synthesis of 10-ethyl-7,8-dihydro-*γ*-ionone (±)-**7**.

Following, we submitted *α*-ionone isomer **3** to epoxidation procedure with *m*-chloroperbenzoic acid to afford separable *cis*/*trans* mixtures of epoxides **4a**/**4b** respectively. The latter compounds subsequently underwent regiospecific hydrogenation using H_2_/Raney Nickel affording **5a**/**5b**. The latter compounds were added to an excess (2.5–3 equiv, −78 °C) of LDA in THF and then warmed at reflux. After quenching, we obtained the *cis*/*trans* mixtures of alcohol **6a**/**6b** respectively showing the same diastereoisomeric ratio as the starting epoxides (*cis*/*trans* 4:1). The obtained allylic alcohols were acetylated and then the acetate group was reductively removed by treatment with triethylammonium formate and palladium catalyst to give 10-ethyl-7,8-dihydro-*γ*-ionone (±)-**7** (Scheme 2).

The above mentioned allylic alcohols **6a/6b** are suitable starting materials for the preparation of enantio enriched isomers **7**. Indeed, we have established that lipase-mediated acetylation of 4-hydroxy *γ*-ionone yield enantiopure (4*R*,6*S*)-4-acetoxy-*γ*-ionone [17]. The reaction proceeds with high enantioselectivity and with complete diastereoselectivity allowing the exclusive transformation of the *cis* isomers.

**Scheme 3:**
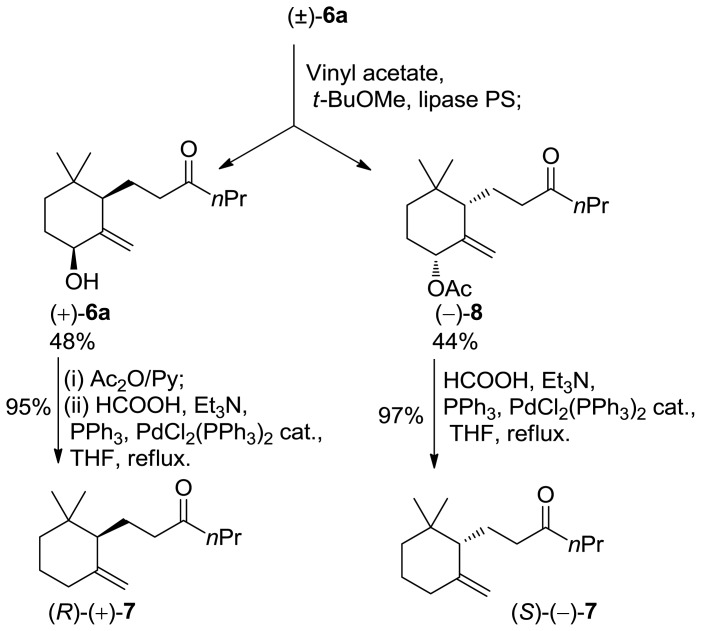
Synthesis of enantio enriched 10-ethyl-7,8-dihydro-*γ*-ionone isomer (+) and (−)-**7**.

Therefore, we performed the enzyme mediated resolution of the above mentioned alcohols (Scheme 2). The described reductive elimination of the acetate group proceeds without racemization allowing the preparation of enantioenriched 10-ethyl-7,8-dihydro-*γ*-ionone isomers. Noteworthy, since the diastereoisomeric allylic alcohols **6a**/**6b** are separable by chromatography, the resolution procedure gives the corresponding acetylated compounds with high *ee* and *de* leaving unreacted alcohols with low *de*. Accordingly, racemic alcohol **6a** was acetylated with vinyl acetate in the presence of lipase PS as a catalyst. The reaction was interrupted at 50% of conversion to give unreacted alcohol (+)-**6a** (99% *de*, 92% *ee*) and acetate (−)-**8** (99% *de*, 99% *ee*). The reductive removal of the acetoxy group converted the latter compounds into 10-ethyl-7,8-dihydro-*γ*-ionone (+)-**7** (92% *ee*) and 10-ethyl-7,8-dihydro-*γ*-ionone (−)-**7** (99% *ee*), respectively (Scheme 3).

### 2.2. Synthesis of 4,5-Didehydro-α-ionone

**Scheme 4:**
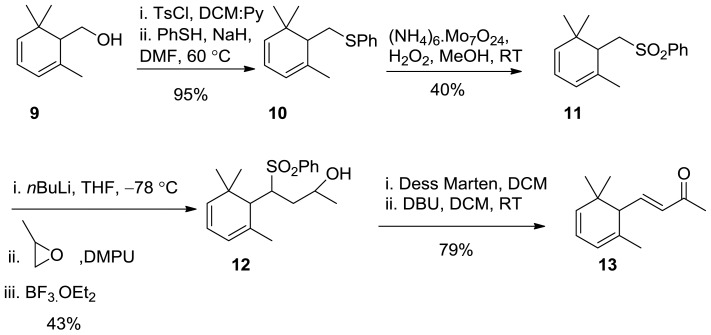
Synthesis of 4,5-didehydro-*α*-ionone **13**.

Aldol condensation of readily available aldehyde with acetone in the presence of base and Wittig reaction with phosphonate were not successful to reach the target compound because isomeric products are inseparable by the usual methodologies. It was found that the readily available alcohol **9** [19] is a good starting material for the synthesis. Tosylation of the free OH-group, followed by addition of thiophenol, deprotonated by NaH afforded the sulphide **10** in 95% yield. Oxidation of the latter compound with a mixture of (NH_4_)_6_.Mo_7_O_24_ and H_2_O_2_ gives the sulphone **11** in moderate yield where the disulfide occurred as a by-product. Sulphone **11** was deprotonated by *n*BuLi at −78 °C, then epoxide in DMPU were added, followed by the addition of BF_3_.OEt_2_ to the reaction mixture. The reaction mixture was stirred for a further 5 h at −78 °C and was left overnight to warm at RT to give **12** in 43% yield that was oxidized by Dess–Martin reagent to give the corresponding ketone in quantitative yield. Removal of PhSO_2_ group by DBU regenerates the target compound **13** (Scheme 4) [20].

## 3. Experimental Section

### 3.1. General Procedures and Instrumentation

All moisture-sensitive reactions were carried out under a static atmosphere of nitrogen. All reagents were of commercial quality. Lipase from *Pseudomonas cepacia* (PS), Amano Pharmaceuticals Co., Japan, 30 units/mg, was employed in this work. TLC: Merk silica gel 60 F_254_ plates. Column chromatography (CC): silica gel. GC-MS analyses: HP-6890 gas chromatograph equipped with a 5973 mass detector, using a HP-5MS column (30 m × 0.25 mm, 0.25 μm firm thickness; Hewlett Packard). Chiral GC analyses: DANI-HT-86.10 gas chromatograph; enantiomer excesses determined on a CHIRASIL DEX CB-Column. Optical rotations: Jasco-DIP-181 digital polarimeter. ^1^H and ^13^C Spectra: CDCl_3_ solution at room temperature; Bruker-AC-400 spectrometer at 400 and 100 MHz, respectively; chemical shifts in ppm relative to internal SiMe_4_ (=0 ppm), *J* values in Hz. IR spectra were recorded on a Perkin-Elmer 2000 FT-IR spectrometer; films; *ν* in cm^−1^. Melting points were measured on a Reichert apparatus, equipped with a Reichert microscope, and are uncorrected. Microanalyses were determined on an analyzer 1106 from Carlo Erba.

### 3.2. Synthesis of (E)-3-(2,6,6-Trimethylcyclohex-2-en-1-yl)prop-2-en-1-ol **2**

**2** was prepared according to reported literature [21] as a colourless oil; ^1^H NMR (400 MHz, CDCl_3_) δ 5.75 (d, *J* = 16.0 Hz, 1H), 5.64 (dt, *J* = 16.0, 6.0 Hz, 1H), 5.45 (d, *J* = 7.0 Hz, 1H), 4.35 (d, *J* = 14.0 Hz, 2H), 2.65 (d, *J* = 6.2 Hz, 1H), 1.75 (s, 3H), 1.65–1.53 (m, 2H), 1.49–1.41 (m, 2H), 1.31 (br s, 1H), 0.98 (s, 6H).

### 3.3. Synthesis of (E)-1-(2,6,6-Trimethylcyclohex-2-en-1-yl)hex-1-en-3-one (**3**)

To a mixture of **2** (25 g, 64.4 mmol) in CHCl_3_ (200 mL), MnO_2_ (30 g, 345 mmol) was added, and the mixture was stirred at reflux for 6 h. The mixture was then cooled, filtered (using filter paper) and the organic phase was concentrated under reduced pressure to afford an oil (13 g). The latter was dissolved in dry diethyl ether (150 mL) and treated under stirring with an excess of *n*-propylmagnesium bromide (100 mL of a 1 M solution in ether) keeping the reaction temperature under 5 °C by external cooling (ice bath). The usual work-up afforded crude carbinol that was dissolved in CHCl_3_ (200 mL) and treated with MnO_2_ (30 g, 345 mmol) stirring at reflux for 12 h. After filtration and concentration, the crude ketone was purified by chromatography (hexane/Et_2_O 95:5) and bulb to bulb distillation (oven temperature 105 °C, 0.4 mmHg) to afford pure **3** (8.4 g, 63% yield), 98% chemical purity (GC)) as a colorless oil;^1^H NMR (400 MHz, CDCl_3_) δ 6.64 (dd, *J* = 15.6, 9.6 Hz, 1H), 6.06 (d, *J* = 15.6 Hz, 1H), 5.48 (bs, 1H), 2.52 (t, *J* = 7.2 Hz, 2H), 2.27 (d, *J* = 10 Hz, 1H), 2.04 (m, 1H), 1.67 (ddd, *J* = 13.4, 6.5, 1.4 Hz, 1H), 1.55 (s, 3H), 1.41 (m, 2H), 1.23 (m, 2H), 0.94 (t, *J* = 1.7 Hz, 3H), 0.93 (s, 3H), 0.82 (s, 3H); ^13^C NMR (100 MHz) δ 212.8, 135.3, 132.6, 121.4, 73.2, 54.5, 40.0, 32.0, 27.9, 27.3, 19.0, 14.3; IR (nujol, cm^−1^) 1684, 1619, 1554. GC-MS *m*/*z:* 221.18 (M^+^, 1); Anal. Calcd for C_15_H_24_O: C, 81.76; H, 10.98.Found: C, 81.75; H, 10.90.

### 3.4. General Procedure for the Synthesis of Adducts **4a/4b**

*m*-Chloroperbenzoic acid (7.4 g, of 75% wet acid, 32 mmol) was added to a solution of racemic *α*-ionone isomer 3 (7 g, 30 mmol) in methylene chloride (75 mL) at 0 °C. The reaction mixture was stirred at 0 °C for 2 h and then filtered in order to remove the *m*-clorobenzoic acid precipitate. The organic phase was washed with saturated Na_2_SO_3_ solution (50 mL) and saturated NaHCO_3_ solution (50 mL), respectively, dried over Na_2_SO_4_ and concentrated under reduced pressure. The residue was chromatographed on a silica gel column (hexane/Et_2_O 9:1) to give the corresponding α-epoxy-derivatives.

Epoxide 4 (85% yield) 4a:4b=4:1 separable by chromatography. Both colorless oil.

#### 3.4.1. (*E*)-1-[(1*R*,2*R*,6*S*)-1,3,3-Trimethyl-7-oxabicyclo[4.1.0]heptan-2-yl]hex-1-en-3-one (**4a**)

^1^H NMR (400 MHz, CDCl_3_) δ 5.45-5.41 (m, 1H), 5.39 (bs, 1H), 5.48 (bs, 1H), 4.10 (bs, 1H), 2.10 (d, *J* = 6.4 Hz, 1H), 2.00 (m, 2H), 1.59 (dd, *J* = 4.4, 1.6 Hz, 1H), 1.54 (m, 2H), 1.40 (m, 2H), 1.37 (s, 3H), 0.94 (td, *J* = 7.2, 1.6 Hz, 3H), 0.93 (s, 3H), 0.81(s, 3H); ^13^C NMR (100 MHz) δ 212.8, 135.3, 132.6, 121.4, 73.2, 54.5, 40.0, 32.0, 27.9, 27.3, 19.0, 14.3; IR (nujol, cm^−1^) 1684; GC-MS *m*/*z*: 237.18 (M^+^, 1); Anal. Calcd for C_15_H_24_O_2_: C, 76.23; H, 10.24. Found: C, 76.34; H, 10.55.

#### 3.4.2. (*E*)-1-[(1*R*,2*S*,6*S*)-1,3,3-Trimethyl-7-Oxabicyclo[4.1.0]heptan-2-yl]hex-1-en-3-One (**4b**)

^1^H NMR (400 MHz, CDCl_3_) δ 5.47-5.43 (m, 1H), 5.41 (bs, 1H), 5.52 (bs, 1H), 4.13 (bs, 1H), 2.14 (d, *J* = 6.4 Hz, 1H), 2.00 (m, 2H), 1.61 (dd, *J* = 4.4, 1.6 Hz, 1H), 1.54 (m, 2H), 1.45 (m, 2H), 1.37 (s, 3H), 0.94 (td, *J* = 7.2, 1.6 Hz, 3H), 0.93 (s, 3H), 0.81(s, 3H); ^13^C NMR (100 MHz) δ 212.4, 135.1, 132.3, 121.1, 73.3, 54.1, 40.2, 32.5, 27.9, 27.3, 19.1, 14.1; IR (nujol, cm^−1^) 1680; GC-MS *m*/*z:* 237.18 (M^+^, 1); Anal. Calcd for C_15_H_24_O_2_: C, 76.23; H, 10.24. Found: C, 76.34; H, 10.55.

### 3.5. General Procedure for the Synthesis of Adducts **5a/5b**

H_2_ (360 mL, 1 equiv) was adsorbed of a solution of **4a** or **4b** (4.5 g, 19mmol, 1 equiv) in EtOAc (150 mL) and excess of Rany-Ni for 2 h. The reaction mixture was filtered off and concentrated under reduced pressure. The residue was chromatographed on a silica gel column (hexane/EtOAc 9:1) to give the corresponding product.

#### 3.5.1. 1-[(1*R*,2*R*,6*S*)-1,3,3-Trimethyl-7-Oxabicyclo[4.1.0]heptan-2-yl]hexan-3-One (**5a**)

(3.4 g, 74% yield, 98% chemical purity (GC)) as a colorless oil; ^1^H NMR (400 MHz, CDCl_3_) δ 2.85 (bs, 1H), 2.67–2.59 (m, 2H), 2.43–2.31 (m, 2H), 1.86–1.74 (m, 3H), 1.68–1.58 (m, 2H), 1.56–1.43 (m, 2H), 1.34–1.27 (m, 2H), 1.24 (s, 3H), 0.86 (t, *J* = 14.8 Hz, 3H), 0.81 (s, 3H), 0.76 (s, 3H); ^13^C NMR (100 MHz) δ 2.11.4, 60.2, 46.5, 45.0, 42.3, 31.8, 28.4, 27.9, 27.5, 27.0, 26.9, 22.3, 21.8, 17.6, 14.1; IR (nujol, cm^−1^) 1684, 1109, 990. GC-MS *m/z*: 239.19 (M^+^, 1); Anal. Calcd for C_15_H_26_O_2_: C, 75.58; H, 10.99. Found: C, 75.55; H, 11.02.

#### 3.5.2. 1-[(1*R*,2*S*,6*S*)-1,3,3-Trimethyl-7-Oxabicyclo[4.1.0]heptan-2-yl]hexan-3-One (**5b**)

^1^H NMR (400 MHz, CDCl_3_) δ 2.83 (bs, 1H), 2.64–2.57 (m, 2H), 2.45–2.33 (m, 2H), 1.83–1.75 (m, 3H), 1.67–1.55 (m, 2H), 1.52–1.45 (m, 2H), 1.32–1.25 (m, 2H), 1.25 (s, 3H), 0.87 (t, *J* = 14.8 Hz, 3H), 0.82 (s, 3H), 0.77 (s, 3H); ^13^C NMR (100 MHz) δ 211.2, 60.3, 46.4, 45.2, 42.2, 31.9, 28.5, 27.9, 27.3, 27.1, 26.9, 22.3, 21.8, 17.6, 14.1; IR (nujol, cm^−1^) 1683, 1109, 990. GC-MS *m*/*z*: 239.19 (M^+^, 1); 55 (15). Anal. Calcd for C_15_H_26_O_2_: C, 75.58; H, 10.99. Found: C, 75.55; H, 11.02.

### 3.6. General Procedure for the Synthesis of Adducts **6a/6b**

*n*BuLi (3.0 mL of a 10 M solution in hexane) was added dropwise to a cooled (−78 °C) solution of *i*Pr_2_NH (5.3 g, 50 mmol) in dry THF (90 mL) under nitrogen. The mixture was stirred at this temperature for 30 min. then a solution of the epoxide **5a** or **5b** (2.5 g, 10 mmol) in dry THF (20 mL) was added dropwise. The reaction was gradually warmed to r.t (1 h) and then was heated under reflux until no more starting epoxide was detected by TLC analysis (3 h). After cooling to room temperature, the mixture was poured into a mixture of crushed ice and 5% HCl solution. (80 mL) and extracted with Et_2_O (3 × 200 mL). The organic phase was successively washed with saturated aqueous NH_4_Cl solution (100 mL), brine, dried over Na_2_SO_4_ and concentrated under reduced pressure. The residue was purified by chromatography (eluting from hexane/AcOEt 9:1 to hexane/AcOEt 1:1) to give allylic alcohol.

#### 3.6.1. 1-((1*R*,5*S*)-5-Hydroxy-2,2-Dimethyl-6-Methylenecyclohexyl)hexan-3-One (**6a**)

**6a** (1.95 g, 82% yield, 98% chemical purity (GC)) as a colorless oil; ^1^H NMR (400 MHz, CDCl_3_) δ 5.16 (bs, 1H), 4.64 (bs, 1H), 3.94 (bs, 1H), 2.56–2.48 (m, 2H), 2.34–2.26 (m, 2H), 1.91–1.81 (m, 2H), 1.75–1.67 (m, 2H), 1.64–1.54 (m, 2H), 1.51–1.39 (m, 2H), 1.29–1.24 (m, 2H), 0.97 (s, 3H), 0.89 (t, *J* = 14.8 Hz, 3H), 0.73 (s, 3H); ^13^C NMR (100 MHz) δ 2.11.4, 60.2, 46.5, 45.0, 42.3, 31.8, 28.4, 27.9, 27.5, 27.0, 26.9, 22.3, 21.8, 17.6, 14.1; IR (nujol, cm^−1^) 3334, 1684, 1619. GC-MS *m*/*z*: 239.19 (M^+^, 1); Anal. Calcd for C_15_H_26_O_2_: C, 75.58; H, 10.99. Found: C, 75.65; H, 10.99.

#### 3.6.2. 1-((1*S*,5*S*)-5-Hydroxy-2,2-Dimethyl-6-Methylenecyclohexyl)hexan-3-One (**6b**)

^1^H NMR (400 MHz, CDCl_3_) δ 5.18 (bs, 1H), 4.66 (bs, 1H), 3.95 (bs, 1H), 2.58–2.47 (m, 2H), 2.33–2.28 (m, 2H), 1.92–1.83 (m, 2H), 1.75–1.67 (m, 2H), 1.64–1.54 (m, 2H), 1.53–1.38 (m, 2H), 1.29–1.24 (m, 2H), 0.98 (s, 3H), 0.90 (t, *J* = 14.8 Hz, 3H), 0.75 (s, 3H); ^13^C NMR (100 MHz) δ 2.11.4, 60.2, 46.5, 45.0, 42.3, 31.8, 28.4, 27.9, 27.5, 27.0, 26.9, 22.3, 21.8, 17.6, 14.1; IR (nujol, cm^−1^) 3335, 1681, 1620. GC-MS *m*/*z*: 239.19 (M^+^, 1); Anal. Calcd for C_15_H_26_O_2_: C, 75.58; H, 10.99. Found: C, 75.65; H, 10.99.

### 3.7. General Procedure for Reduction of Allylic Alcohol **6a** to 10-Ethyl-7,8-dihydro-γ-ionone Isomers **7**

A sample of compound **6a** (500 mg, 2.0 mmol) was converted into the corresponding acetate by treatment with pyridine (5 mL) and Ac_2_O (5 mL) at room temperature for 24 h. The crude product was added to a solution of formic acid (180 mg, 4.0 mmol), Et_3_N (440 mg, 4.4 mmol), (PPh_3_)_2_PdCl_2_ (41 mg, 0.06 mmol) and triphenylphosphine (75 mg, 0.3 mmol) in dry THF (20 mL). The mixture was refluxed under a static nitrogen atmosphere until reduction was complete (2 h, TLC analysis). The reaction was then diluted with ether (30 mL) and washed with water (20 mL), 5% HCl solution (20 mL), saturated aqueous NaHCO_3_ solution (20 mL), and brine. The organic phase was dried (Na_2_SO_4_) and concentrated under reduced pressure. The residue was purified by chromatography (hexane/Et_2_O 95:5) and bulb-to-bulb distillation to give *γ*-ionone isomers **7** (84% yield, 97% isomeric purity).

10-Ethyl-7,8-dihydro-*γ*-ionone (±)-**7** (99% chemical purity (GC)) as a colorless oil; ^1^H NMR (400 MHz, CDCl_3_) δ 4.75 (bs, 1H), 4.50 (d, *J* = 2.4 Hz,1H), 2.38–2.30 (m, 2H), 2.27–2.18(m, 2H), 2.05–1.94(m, 2H), 1.84–1.76(m, 3H), 1.70–1.55 (m, 2H), 1.54–1.46 (m, 2H), 1.30–1.17 (m, 2H), 0.91 (s, 3H), 0.89 (t, *J* = 7.2 Hz, 3H), 0.86 (s, 3H); ^13^C NMR (100 MHz) δ 211.8, 149.5, 109.7, 53.9, 45.3, 41.7, 36.2, 35.2, 32.5, 28.6, 26.8, 23.9, 20.7, 17.7, 14.4; IR (nujol, cm^−1^) 1684, 1619. GC-MS *m*/*z* (rel intensity) 223.20 (M^+^, 1); Anal. Calcd for C_15_H_26_O: C, 81.02; H, 11.79. Found: C, 81.14; H, 11.67.

### 3.8. Lipase-Mediated Resolution of Alcohols **6a**

Diastereoisomerically pure alcohol **6a** (obtained from epoxide **5a**) was employed in the resolution procedure. A sample of the above-mentioned racemic material (5 g, 22.5 mmol), lipase PS (5 g), vinyl acetate (25 mL) and *t*BuOMe (100 mL) was stirred at room temperature, and the formation of the acetate was monitored by TLC analysis. The reaction was stopped at about 50% of conversion of **6a** into (−)-**8**. The enzyme was then filtered, and the solvent was evaporated at reduced pressure after which the residue was purified by chromatography (eluting from hexane/AcOEt 9:1 to hexane/AcOEt 1:1). The first-eluted fractions afforded derivatives (−)-**8** (45% yield). The last eluted fractions afforded derivatives (+)-**6a** (49% yield).

#### 3.8.1. (1*S*,3*R*)-4,4-Dimethyl-2-Methylene-3-(3-Oxohexyl)cyclohexyl Acetate (−)-**8**

(Colorless oil; 99% chemical purity, 99% *de* (GC); 99% *ee* (chiral GC); [α]^24^_D_= −11.73° (*c =* 1.0 g/dL, CHCl_3_); ^1^H NMR (400 MHz, CDCl_3_) δ 5.08 (bs, 1H), 4.67 (bs, 1H), 2.47–2.39 (m, 2H), 2.34–2.26 (m, 2H), 2.02 (s, 3H), 1.88–1.80 (m, 3H), 1.75–1.64 (m, 2H), 1.61–1.54 (m, 2H), 1.37–1.30 (m, 2H), 1.26–1.23 (m, 2H), 0.97 (s, 3H), 0.89 (t, *J* = 14.8 Hz, 3H), 0.80 (s, 3H); ^13^C NMR (100 MHz) δ 2.11.4, 170.3, 75.2, 60.7, 52.0, 45.3, 41.6, 30.0, 29.8, 21.6, 20.4, 17.7, 14.5, 14.1; IR (nujol, cm^−1^) 1684, 1619. GC-MS *m*/*z* (rel intensity) 281.20 (M^+^, 1); Anal. Calcd for C_17_H_28_O_3_: C, 72.82; H, 10.06. Found: C, 72.85; H, 10.11.

#### 3.8.2. (1*S*,3*R*)-4,4-Dimethyl-2-methylene-3-(3-oxohexyl)cyclohexyl Acetate (+)-**8**

(Colorless oil; 99% chemical purity, 99% *de* (GC); 92% *ee* (chiral GC); [*α*]^24^_D_= +8.47° (*c =* 1.0 g/dL, CHCl_3_); IR, ^1^H NMR, MS: in accordance with that of (−)-**8.**

### 3.9. Synthesis of Enantioenriched 10-Ethyl-7,8-dihydro-γ-ionone Isomers

The above obtained compounds (−)-**8**, (+)-**8** were submitted to the reductive deoxygenation procedure described above in Section 3.7 to afford *10-ethyl-7*,*8-dihydro-γ-ionone isomers* (−)-**7**, (+)-**7** respectively. The latter compounds showed the following analytical data.

#### 3.9.1. (*S*)-1-(2,2-Dimethyl-6-Methylenecyclohexyl)hexan-3-One (−)-**7**

(Colorless oil, 99% chemical purity, 97% regioisomeric purity (chiral GC); [*α*]^24^_D_ = −20.2° (*c* = 1.0 g/dL, CHCl_3_); IR, ^1^H NMR, MS: in accordance with that of (±)-**7**.

#### 3.9.2. (*R*)-1-(2,2-Dimethyl-6-methylenecyclohexyl)hexan-3-one (+)-**7**

(Colorless oil; 99% chemical purity, 94% regioisomeric purity (chiral GC); [*α*]^24^_D_= +17.3° (*c* = 1.0 g/dL, CHCl_3_); IR, ^1^H NMR, MS: in accordance with that of (±)-**7**.

### 3.10. Synthesis of Phenyl((2,6,6-Trimethylcyclohexa-2,4-Dien-1-yl)methyl)Sulfane **10**

In a 100 mL round bottom flask, **9** (3.5 g, 2.3 mmol) was dissolved in a mixture of Py:DCM (1:1, 12 mL). TsCl (5.7 g, 3 mmol, 1.3 equiv) was added into reaction mixture and stirred at room temperature for 1.5 h. Water was added and extracted with Et_2_O (3 × 100 mL); the organic phase was washed with sat. NaHCO_3_, then 5% HCL, brine, dried over Na_2_SO_4_, and concentrated under reduced pressure, and used for the next step without further purification in quantitative yield.

In a round bottom flask, thiophenol (2.6 g, 23.54mmol, 1.2 equiv) was dissolved in DMF (30 mL), NaH (1.4 g 60%, 3 equiv) was added portion wise. After 15 min, TsCl (6.2 g, 19.62 mmol) was added. The reaction mixture was stirred at room temperature for 1 h, then at 60 °C for 2.5 h, then cooled to room temperature 0.1 N NaOH (30 mL) was added and extracted with Et_2_O, the organic phase was washed with water, brine, dried over Na_2_SO_4_, and concentrated under reduced pressure, and the crude was purified by CC using *n*hexane:EtOAc 100:2; 4.8 g, 95% yield.

^1^H NMR (400 MHz, CDCl_3_) δ 7.41–7.23 (m, 5H), 5.20–5.87 (m, 3H), 3.58–3.95 (m, 2H), 2.8 (t, *J* = 10.4Hz, 1H), 1.85 (s, 3H), 1.04 (s, 3H), 0.92 (s, 3H); ^13^C NMR (100 MHz) δ 155.8, 154.9, 153.5, 128.9, 125.4, 117.6, 114.7, 53.3, 38.7, 33.2, 24.4, 22.3 IR (nujol, cm^−1^) 1680, 1606, 1454, 1100, 990. GC-MS *m*/*z*: 245.13 (M^+^, 1); Anal. Calcd for C_16_H_20_S: C, 78.63; H, 8.25. Found: C, 78.65; H, 8.35.

### 3.11. Synthesis of (((2,6,6-Trimethylcyclohexa-2,4-dien-1-yl)Methyl)Sulfonyl)Benzene (**11**)

In a round bottom flask, **10** (4.5 g, 18.44 mmol) was dissolved in MeOH (30 mL) and cooled at 0 °C. H_2_O_2_ (4 mL of 30%), and (NH_4_)_2_MoO_4_ (5.4 g, 22.12 mmol, 1.2 equiv) were added. The reaction mixture was warmed to room temperature, and stirred overnight. Na_2_S_2_O_5_ (solid ~ calculated 5 g) was added in order to remove the excess oxidant. Extracted with DCM, dried over Na_2_SO_4_, and concentrated under reduced pressure, and the crude was purified by CC using *n*hexane:EtOAc 100:10; 1.15 g, 40% yield.^1^H NMR (400 MHz, CDCl_3_) δ 7.43–7.25 (m, 5H), 5.89–5.21 (m, 3H), 3.98–3.68 (m, 2H), 2.8 (t, *J* = 10.4Hz, 1H), 1.86 (s, 3H), 1.05 (s, 3H), 0.95 (s, 3H); ^13^C NMR (100 MHz) δ 155.8, 154.9, 153.5, 128.9, 125.4, 117.6, 114.7, 53.3, 38.7, 33.2, 24.4, 22.3 IR (nujol, cm^−1^) 1680, 1606, 1454, 1100, 990. GC-MS *m*/*z*: 277.12 (M^+^, 1); Anal. Calcd for C_16_H_20_O_2_S: C, 69.53; H, 11.58. Found: C, 69.54; H, 11.60.

### 3.12. Synthesis of 4-(Phenylsulfonyl)-4-(2,6,6-Trimethylcyclohexa-2,4-Dien-1-yl)butan-2-ol (**12**)

In a three neck 100 mL round bottom flask, *n*BuLi (0.4 mL of a 10 M solution in hexane) was added dropwise to a cooled (−78 °C) solution of **11** (1.15 g, 4.16 mmol) in dry THF (25 mL) under nitrogen, then stirred for 30 min. Subsequently, a mixture of propylene epoxide (1.6 mL) in DMPU [1,3-Dimethyl-3,4,5,6-tetrahydro-2(1H)-pyrimidinone] (5 mL) was added, then BF_3_OEt_2_ (3 mL) was added and the reaction mixture was stirred for 5 h at −78 °C. The reaction mixture was allowed to warm up at room temperature and stirred overnight. Water (100 mL) was added and extracted with DCM (3 × 100 mL); the organic phase was washed with brine, dried over Na_2_SO_4_, and concentrated under reduced pressure, and the crude was purified by CC using *n*hexane:EtOAc 10:3 to 5:5 to afford **12**. (N.B: recovered starting material 500 mg)

(600 mg, 43% yield, 98% chemical purity (GC)) as a colorless oil; ^1^H NMR (400 MHz, CDCl_3_) δ: 7.84–7.82 (m, 2h, Ph), 7.58–7.48 (m, 3h, Ph), 5.81 (d, *J* = 15.8 Hz, 1H), 5.67 (d, *J* = 15.8 Hz, 1H), 5.35–5.25(m, 1H), 3.77 (m, 1H), 3.59 (bs, 1H), 3.11(m, 1H), 2.54(d, *J* = 15.8 Hz, 1H), 2.28(t, *J* = 4.8 Hz, 1H), 2.09(s, 3H), 1.31 (bs, 3H), 1.29 (bs, 3H), 0.95 (d, *J* = 6 Hz, 1H) 0.89 (s, 3H); ^13^C NMR (100 MHz) δ 153.9, 144.7, 137.7, 133.8, 129.9, 123.4, 117.9, 64.8, 57.9, 45.1, 36.7, 25.2, 23.5; IR (nujol, cm^−1^) 3360, 1684, 1619, 1454, 1109, 990. GC-MS *m*/*z*: 335.16 (M^+^, 1); Anal. Calcd for C_19_H_26_O_3_S: C, 68.23; H, 7.84; S, 9.59. Found: C, 68.35; H, 7.85; S, 9.62.

### 3.13. Synthesis of (E)-4-(2,6,6-Trimethylcyclohexa-2,4-Dien-1-yl)but-3-en-2-One (**13**)

In a round bottom flask, **12** (0.5 g, 0.15 mmol) was dissolved in DCM (10 mL). Dess Martin (1.3 equiv, 0.82 g, 0.19 mmol) was added portion wise. After the reaction was stirred at room temperature for 2 h, Na_2_S_2_O_5_ (solid~calculated 0.5 g) was added in order to remove the excess oxidant. Extracted with DCM. The organic phase was washed with water, brine, dried over Na_2_SO_4_, and concentrated under reduced pressure. The crude was dissolved in DCM (5 mL), and DBU (0.1 mL) was added. The reaction was stirred at room temperature for 30 min, and the reaction mixture was washed with water, brine, dried over Na_2_SO_4_, and concentrated under reduced pressure. The crude was purified by CC using EtOAc: *n*hexane 7:3.

(0.15 g, 79% yield, 98% chemical purity (GC)) as a colorless oil: ^1^H NMR (400 MHz, CDCl_3_) δ 6.65 (dd, *J* = 15.6, 9.6 Hz, 1H), 6.06 (d, *J* = 15.6 Hz, 1H), 5.89–5.21 (m, 3H), 2.8 (t, *J* = 10.4Hz, 1H), 2.27(s, 3H), 1.86 (s, 3H), 1.05 (s, 3H), 0.95 (s, 3H); ^13^C NMR (100 MHz) δ 206.9, 156.2, 144.7, 133.7, 119.7, 117.9, 60.5 30.6, 28.3, 26.2. IR (nujol, cm^−1^) 1684, 1619, 1454, 1109, 990. GC-MS *m*/*z*: 191.14 (M^+^, 1); Anal. Calcd for C_13_H_18_O: C, 82.06; H, 9.53. Found: C, 82.11; H, 9.54.

## 4. Conclusions

A new stereospecific approach to the 10-ethyl-7,8-dihydro-*γ*-ionone isomers and 4,5-didehydroionone isomers are described. The 10-ethyl-7,8-dihydro-*γ*-ionone isomers (*R*)-(+)-**7**, and (*S*)-(−)-**7** were prepared starting from commercially available α-ionone **1** in a few regioselective steps. Enantiomer-enriched (*R*)-(+)-**7**, and (*S*)-(−)-**7**were prepared by means of diastereoselective and enantioselective lipase-mediated acetylation of the racemic intermediate 4-hydroxy-*γ*-ionone derivative **6a/6b** followed by a number of chemoselective and regioselective reactions. Moreover, the resolution step confirms the utility of the enzymatic approach to the preparation of enantiomer-enriched norterpenoid odorants.
